# Supplementary material for: Modulation of Gamma Spectral Amplitude and Connectivity During Reaching Predicts Peak Velocity and Movement Duration
Source: Front Neurosci. 2022 Feb 24;16:836703. doi: 10.3389/fnins.2022.836703 (PMC8908429; doi:10.3389/fnins.2022.836703)
Supplement: Supplementary file 1 [file Data_Sheet_1.PDF]

		RT	MT	PV Time	PV Amplitude	Movement Extent
**Distance**	**df**	2 122.00	1.30 79.03	1.58 96.53	1.17 71.46	1.75 106.89
	**F**	24.26	285.52	305.80	868.72	5496.98
	**p**	**< 0.001**	**< 0.001**	**< 0.001**	**< 0.001**	**< 0.001**
	**η^2p**	0.29	0.82	0.83	0.93	0.99
**Short vs Medium**	**μ diff**	7.52	-22.44	-12.65	-17.85	-3.07
	**SE**	1.19	1.42	0.77	0.57	0.05
	**p**	**< 0.001**	**< 0.001**	**< 0.001**	**< 0.001**	**< 0.001**
	**CI**	4.60 10.43	-25.94 -18.94	-14.56 -10.75	-19.26 -16.43	-3.18 -2.95
**Short vs Long**	**μ diff**	7.21	-37.78	-21.09	-32.15	-5.75
	**SE**	1.22	2.08	1.05	1.05	0.06
	**p**	**< 0.001**	**< 0.001**	**< 0.001**	**< 0.001**	**< 0.001**
	**CI**	4.22 10.21	-42.91 -32.65	-23.69 -18.49	-34.73 -29.57	-5.91 -5.59
**Medium vs Long**	**μ diff**	-0.30	-15.34	-8.43	-14.31	-2.68
	**SE**	1.26	1.11	0.71	0.60	0.05
	**p**	1.00	**< 0.001**	**< 0.001**	**< 0.001**	**< 0.001**
	**CI**	-3.41 2.80	-18.07 -12.61	-10.18 -6.68	-15.79 -12.82	-2.81 -2.55

Supplementary Table 1. Results of repeated measure ANOVAs on the effect of target distance on the behavioral indices. RT: Reaction Time; MT: Movement Time; PV: Peak Velocity; μ diff: mean difference; SE: Standard Error of the mean; CI: Confidence Interval; η^2p: partial eta squared.

	FE	Beta	CI		t	p_t	R^2adj	BIC	TLRT	p_{TLRT}
**Model with random intercept**	PV	0.99	0.98	1.0	204	**< 0.0001**	0.92	1792.7	-	-
	MT	0.63	0.62	0.65	93	**< 0.0001**				
**Model with random intercept & slope**	PV	1.06	1.01	1.1	2193	**< 0.0001**	0.95	-174.5	2009	**< 0.0001**
	MT	0.70	0.65	0.75	905	**< 0.0001**				

Supplementary Table 2. Linear mixed-effects regression models on kinematic data. (Top) Mixed-effect regression model including a random intercept for each subject; (Bottom) Mixed-effect regression model including random intercept and slope. FE: Fixed-effects included in the models (PV: Peak Velocity amplitude; MT: Movement Time); Beta: Estimate of the slope for the FE; t= t test; CI: Confidence Interval of the FE estimate; BIC: Bayesian information criterion; R^2adj: adjusted coefficient of determination; TLRT: Theoretical Likelihood Ratio Test. p_t: p value associated to the t statistic testing the effect of the FE; $p_{TLRT:}$ p value resulting from the TLRT which tested which model showed a better fitting of the data. Significant results are reported in bold (alpha=0.05).

Supplementary Table 3.

	F	*Beta*	*SE*	*t*	*p*	*R^2adj*
A. Peak velocity out movement						
All Gamma (25.5-80 Hz)	12.8	0.43	0.12	3.57	**0.0007**	0.17
High Gamma (55.5-80 Hz)	11.2	0.40	0.12	3.34	**0.0015**	0.15
B. Peak velocity back movement						
All gamma (25.5-80 Hz)	1.87	0.18	0.13	1.37	0.18	0.015
High Gamma (55.5-80 Hz)	2.52	0.21	0.13	1.59	0.12	0.026

Supplemental Table 3. Linear regression models of z-transformed peak velocity and gamma amplitudes for both out and back movements.
Differently from the results displayed in Table 1, here we report linear regression modelling of peak velocity (PV) and gamma amplitude at the time of the peak velocity of both the out and back movements. F: F-statistic vs. constant model; Beta: coefficient estimate for the term in the model; SE: Standard Error of the coefficient; t: t statistic testing the null hypothesis that the corresponding coefficient is zero, p: p value; R^2adj: adjusted coefficient of determination (df=57). In bold are reported the significant p values (alpha=0.05).

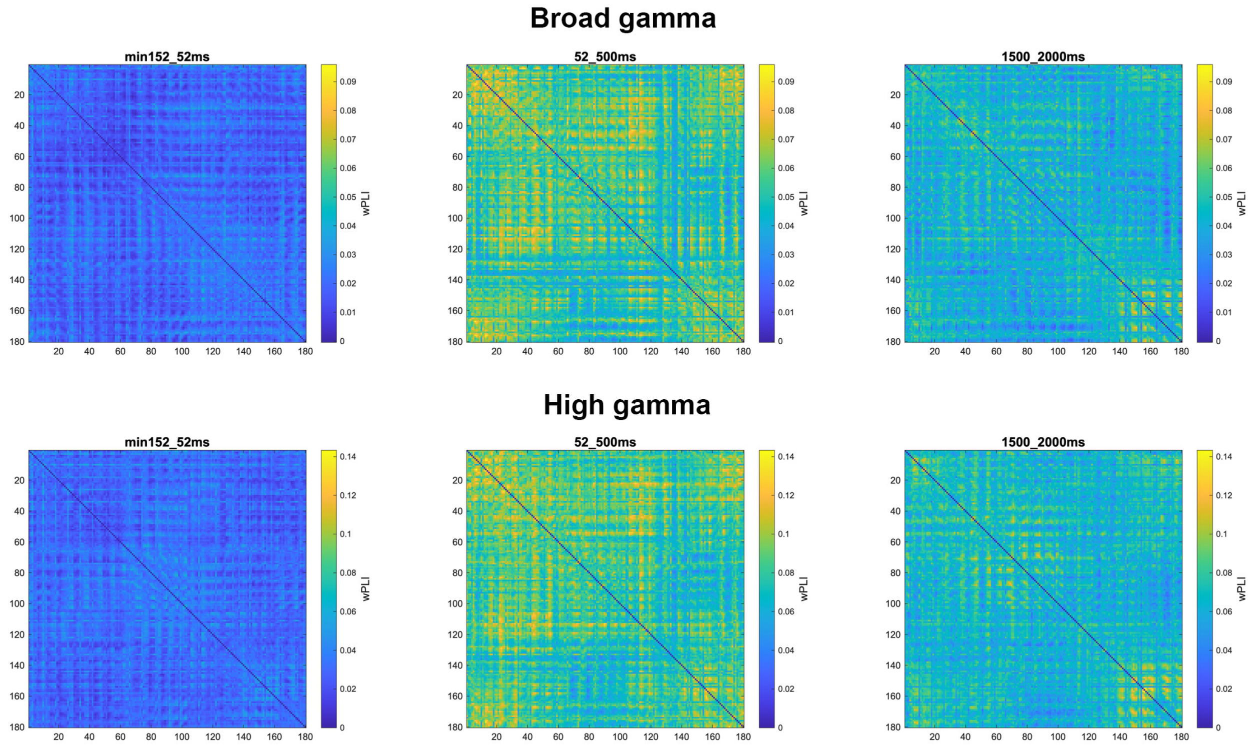

Supplementary Figure 1. Functional connectivity matrices averaged across all participants during movement planning (from 152 to 52ms before movement onset), execution (from 52 to 500ms after movement onset), and post-movement (from 1500 to 2000ms after movement onset) windows for the broad gamma and high gamma frequency band.

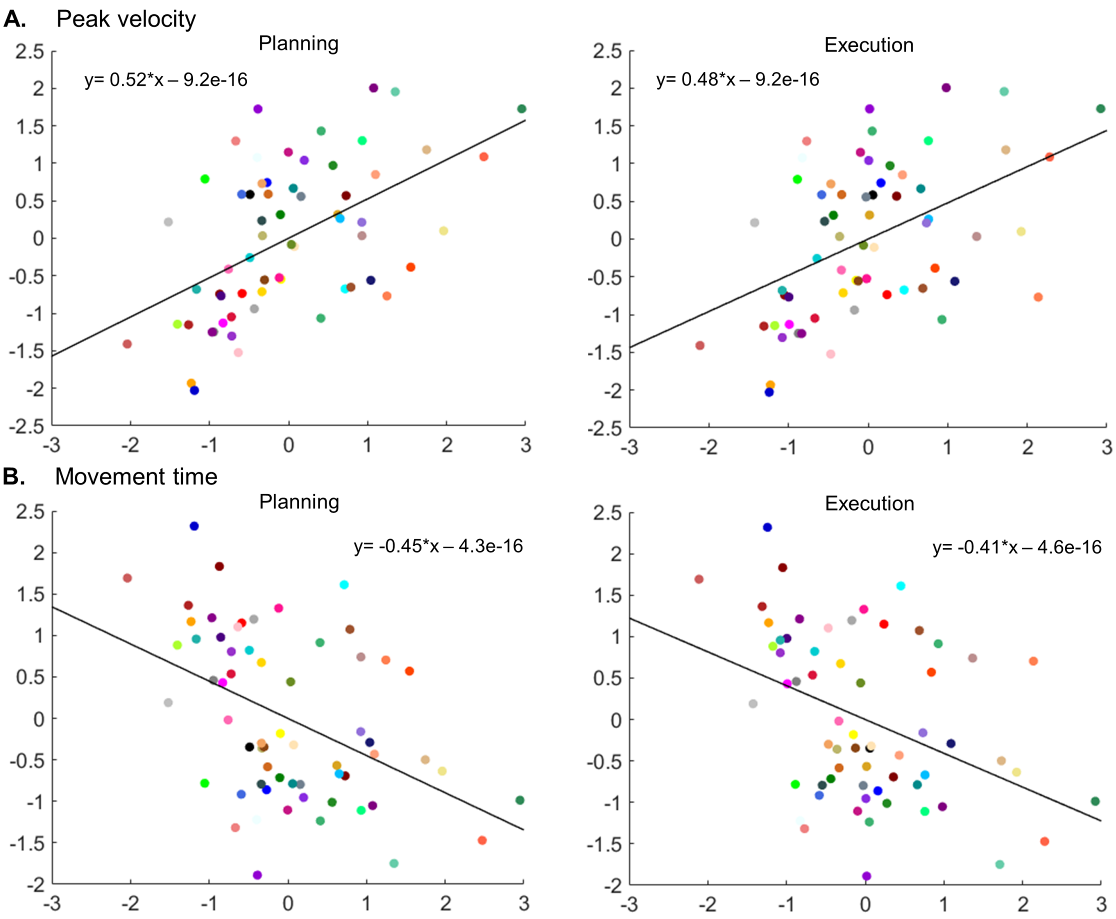

Supplementary Figure 2. Relationship between the z-transformed gamma power (25.5-80 Hz) during the planning and movement time-windows and A. peak velocity amplitude and B. Movement time. Solid line represents the linear fit of the data.
